# Evaluating the efficacy of vatiquinone in preclinical models of Leigh syndrome and GPX4 deficiency

**DOI:** 10.1186/s13023-025-03582-x

**Published:** 2025-02-10

**Authors:** Ernst-Bernhard Kayser, Michael Mulholland, Elizaveta A. Olkhova, Yihan Chen, Holly Coulson, Owen Cairns, Vivian Truong, Katerina James, Brittany M. Johnson, Allison Hanaford, Simon C. Johnson

**Affiliations:** 1 Center for Integrative Brain Research, Seattle Children’s Research Institute, Seattle, USA; 2 Department of Anesthesia and Pain Medicine, University of Washington, Seattle, USA; 3 Department of Laboratory Medicine and Pathology, University of Washington, Seattle, USA; 4 Department of Neurology, University of Washington, Seattle, USA; 5 Department of Applied Sciences, Translational Bioscience, Northumbria University, Newcastle, UK

**Keywords:** Mitochondrial disease, Antioxidant, Vatiquinone, EPI-743, ROS, Leigh syndrome, GPX4

## Abstract

**Background:**

Genetic mitochondrial diseases are a major challenge in modern medicine. These impact ~ 1:4,000 individuals and there are currently no effective therapies. Leigh syndrome is the most common pediatric presentation of mitochondrial disease. In humans, patients are often treated with antioxidants, vitamins, and strategies targeting energetics. The vitamin-E related compound vatiquinone (EPI-743, α-tocotrienol quinone) has been the subject of at least 19 clinical trials in the US since 2012, but the effects of vatiquinone on an animal model of mitochondrial disease have not yet been reported. Here, assessed the impact of vatiquinone in cellular assays and animal models of mitochondrial disease.

**Methods:**

The efficacy of vatiquinone in vitro was assessed using human fibroblasts and HEK293 cells treated with the ferroptosis inducers RSL3 and BSO + Fe(III)Citrate, the mitochondrial oxidative stress inducer paraquat, and the electron transport chain complex I inhibitor rotenone. The therapeutic potential of vatiquinone in vivo was assessed using the tamoxifen-induced mouse model for GPX4 deficiency and the *Ndufs4* knockout mouse model of Leigh syndrome.

**Results:**

Vatiquinone robustly prevented death in cultured cells induced by RSL3 or BSO/iron, but had no effect on paraquat induced cell death. Vatiquinone had no impact on disease onset, progression, or survival in either the tamoxifen-inducible GPX4 deficient model or the *Ndufs4*(-/-) mouse model, though the drug may have reduced seizure risk.

**Conclusions:**

Vatiquinone prevents ferroptosis, but fails to attenuate cell death induced by paraquat or rotenone and provided no significant benefit to survival in two mouse models of disease. Vatiquinone may prevent seizures in the *Ndufs4*(-/-) model. Our findings are consistent with recent press statements regarding clinical trial results and have implications for drug trial design and reporting in patients with rare diseases.

## Introduction

Mitochondrial diseases, disorders caused by defects in genes encoding mitochondrial factors, are a major challenge in human medicine. As a group, mitochondrial diseases are the most common cause of inborn errors of metabolism, and a major genetic cause of neurologic disease. Mitochondrial diseases are estimated to impact approximately 1 in 4,000 individuals, though this may underestimate actual cases due to challenges in diagnosis.

Mitochondrial diseases are a clinically and genetically diverse group of disorders: over 400 unique genes have now been linked to mitochondrial disease [1]. Mitochondrial disease can be caused by defects in genes that directly impact mitochondrial electron transport chain (ETC) subunits, genes encoding products peripherally involved in ETC function, genes coding for enzymes involved in maintaining mitochondrial membranes, or genes involved in other mitochondrial processes such as antioxidant defenses.

Over a dozen distinct named clinical syndromes can result from genetic defects in mitochondrial components. These can involve single organ dysfunction, as in optic nerve pathology in Leber’s Hereditary Optic Neuropathy (LHON), or occur as complex multi-system disorders such as Leigh syndrome [2, 3]. Leigh syndrome, or subacute necrotizing encephalopathy, is one of the more severe clinical presentations of mitochondrial disease and the most common pediatric presentation. Leigh syndrome is characterized by early post-natal onset (median ~ 2 years, though adult onset can occur) with muscle weakness, failure to thrive, metabolic dysfunction including hyperlactatemia, and symmetric progressive necrotizing lesions in brain areas including the basal ganglion, and seizures. The CNS lesions are a defining feature of the disease, drive severe neurologic defects, and typically lead to mortality from respiratory failure. Leigh syndrome is genetically heterogeneous: defects in over 110 unique genes have been identified as causes of Leigh syndrome [3].

Defects in genes related to iron homeostasis and antioxidant systems are frequently associated with mitochondrial dysfunction, but inconsistently included under the umbrella of ‘mitochondrial disease.’ For example, Friedreich’s ataxia (FA), caused by defects in the mitochondrial iron homeostasis factor frataxin, and Sedaghatian-type spinal metaphyseal dysplasia (SSMP), caused by defects in glutathione peroxidase 4 (GPX4), are not universally included in ‘mitochondrial disease’ gene lists, though mitochondrial function is impacted by defects in these genes [4–6]. In the case of GPX4, nuclear, cytoplasmic, and mitochondrial isoforms have been characterized, but the roles of each in human disease remain poorly understood [7].

Clinically effective treatments do not yet exist for genetic mitochondrial diseases. Disease management is generally limited to treating symptoms and providing supportive care. Targeted supplementation is often used where indicated, such as CoQ in CoQ deficiencies or nucleosides in MNGIE/TK2 patients, with mixed results [8–10]. More broadly, mitochondrial disease patients are often given vitamins, antioxidants, and/or nutritional supplements, often in a combination known as the ‘mito-cocktail’ [11]. While these approaches do not appear to substantially alter disease course in the majority of cases, drugs putatively targeting oxidative stress and energetics have been the subject of many clinical trials in mitochondrial disease.

Vatiquinone, previously known as EPI-743, is a vitamin E derived compound under development by PTC Therapeutics, acquired from BioElectron Technology Corporation (formerly Edison Pharmaceuticals) in 2019. Vatiquinone was identified in a screen using glutathione-depletion to induce cell death and has been shown to robustly suppress the cell death pathway ferroptosis [12]. The mechanistic target of vatiquinone has been identified as 15-lipoxygenase (15-LO), an enzyme which drives iron-catalyzed lipid peroxidation when reactive oxygen species (ROS) are high. 15-LO generated peroxidated lipids act as a signal for ferroptotic cell death [12, 13]. This cell death pathway is inhibited by glutathione peroxidase 4 (GPX4), which can directly remove membrane phospholipid hydroperoxides [14, 15].

At least 19 human trials of vatiquinone have been initiated, mainly targeting populations with genetic mitochondrial diseases including Leigh syndrome, FA, LHON, and mitochondrial disease as a group (see Table 1, *Discussion*). Cell-based endpoints provided the rationale for these many trials, but, remarkably, no studies have assessed the potential efficacy of vatiquinone in vivo using an animal model of mitochondrial disease, and positive cellular data has been largely restricted to inhibition of ferroptosis. Given the limited evidence for a mechanistic overlap between ferroptosis and mitochondrial disease pathobiology, as well as the lack of in vivo evidence, we sought here to directly assess the efficacy of vatiquinone in vitro and in vivo.

**Table 1:** Clinical trials of vatiquinone

ClinicalTrials ID	Name	Type	Population	Start date	Completion date	Patients enrolled^*^	Results
NCT05485987	A Study of Vatiquinone for the Treatment of Participants With Friedreich Ataxia	Phase 2	FA patients under the age of 7	13/10/2022	05/10/2024	5	n/a (active)
NCT05218655	A Safety Study for Previously Treated Vatiquinone (PTC743) Participants With Inherited Mitochondrial Disease	Phase 3	Subjects With Inherited Mitochondrial Disease	21/06/2022	31/03/2025	100	n/a (active)
NCT05515536	A Study to Assess the Safety and Efficacy of Vatiquinone in Participants With Friedreich Ataxia	Phase 3	Patients With Friedreich Ataxia	08/12/2022	31/12/2027	140	n/a (active)
NCT04378075	Efficacy and Safety Study of Vatiquinone for the Treatment of Mitochondrial Disease Subjects With Refractory Epilepsy	Phase 2, Phase 3	Genetically confirmed mitochondrial disease patients with treatment refractory epilepsy, < 20 years of age	28/09/2020	27/12/2023	60	Not yet posted to Clintrials.gov; press release by PTC Therapeutics June 2023 reported failure to achieve primary endpoint.
NCT04577352	A Study to Assess the Efficacy and Safety of Vatiquinone for the Treatment of Participants With Friedreich Ataxia (MOVE-FA)	Phase 2, Phase 3	Patients with FA. Primary endpoint in patients between 7 and 21.	17/12/2020	02/10/2023	146	Not yet posted to Clintrials.gov; press release by PTC Therapeutics May 2023 reported failure to achieve primary endpoint.
NCT01923584	A Phase 2 A Trial of EPI-743 for Patients With Parkinson’s Disease	Phase 2	Patients with Parkinson’s disease	2013-09	2016-06	15	Not reported
NCT02352896	Long-Term Safety and Efficacy Evaluation of EPI-743 in Children With Leigh Syndrome	Phase 2	Patients with Leigh syndrome	31/01/2014	30/10/2023	30	Not reported
NCT01962363	EPI-743 in Friedreich’s Ataxia Point Mutations	Phase 2	Patients with FA	2013-10	2016-06	4	Not yet posted to Clintrials.gov; press release suggested possible benefits, but patient number was limited.
NCT02104336	Phase 2 Study of EPI-743 in Children With Pearson Syndrome	Phase 2	Children (< 18 years old) with Pearson syndrome	31/08/2014	29/02/2016	2	Terminated (Results from other studies did not support continuation of this trial)
NCT02300753	Emergency Administration of EPI-743 to a Single Patient With Leber’s Hereditary Optic Neuropathy [LHON]	Expanded Access	Patients with LHON				n/a
NCT01370447	EPI-743 for Mitochondrial Respiratory Chain Diseases	Expanded Access	Acutely ill patients with severe mitochondrial respiratory chain disease within 90 days of end-of-life care				n/a
NCT02226458	An Exploratory Open Label Study of EPI-743 (Vincerinone TM) in Children With Autism Spectrum Disorder	Phase 2	Patients with Autism	31/10/2014	31/01/2016	0	Withdrawn
NCT01719523	Open-Trial of EPI-743 for Adults With Tourette Syndrome	Phase 1	Adults with Tourette syndrome	2012-10	2013-10	10	No
NCT01728064	Safety and Efficacy of EPI-743 in Patients With Friedreich’s Ataxia	Phase 2	18–45 year old patients with FA	31/12/2012	29/02/2016	60	Not yet posted to Clintrials.gov. Positive findings claimed in a 2017 review via personal correspondence (*5*)
NCT02257983	Protective Effects of EPI-743 on Noise-Induced Hearing Loss	Phase 2	Healthy adults age 18–30	31/10/2014	29/02/2016	77	No
NCT01721733	Safety and Efficacy Study of EPI-743 in Children With Leigh Syndrome	Phase 2	6 to 17 year old children with clinical and MRI diagnosis of Leigh syndrome	31/10/2012	31/05/2015	35	No
NCT01822249	Phase 2 Study of EPI-743 for Treatment of Rett Syndrome	Phase 2	Up to 18 years old, female, diagnosed with Rett syndrome	2013-01	2014-01	24	Not posted to Clintrials.gov, but reported to have failed primary outcomes (*5*).
NCT01642056	EPI-743 for Metabolism or Mitochondrial Disorders	Phase 1, Phase 2	Children 2–11 years old with neuromuscular disease associated with mitochondrial impairment	01/09/2012	24/09/2019	20	Yes; no evidence of efficacy.
NCT01793090	EPI-743 in Cobalamin C Defect: Effects on Visual and Neurological Impairment	Phase 2	Children 1–20 years old with genetically confirmed Cbl-c defects	2013-01	02/2017	30	No.

Cells empty where data are not available on ClinTrials.gov. *– estimated or real

## Methods

### Vatiquinone

Vatiquinone was purchased commercially from MedKoo Biosciences (Morrisville, NC). At room temperature, vatiquinone is a viscous liquid. For cell culture studies, vatiquinone stock solutions were prepared by dissolving vatiquinone in DMSO. For animal treatments by IP injection, vatiquinone was dissolved in sterile sunflower seed oil or 95% sunflower seed oil with 5% ethanol when co-injected with tamoxifen (details below). In each case, stock solutions were aliquoted and stored at -20º C or -80º C, protected from light, until warmed for injection.

### Cultured fibroblasts and HEK293 cells

Human dermal neonatal fibroblasts (Sigma 106–05 N) and HEK293 cells (Sigma 85120602) were propagated in low-glucose DMEM (D5523) supplemented with 10% fetal bovine serum (FBS, FisherScientific UK 11570506), 100 U/mL penicillin & streptomycin (ThermoFisher 15140122), and D-glucose (Sigma 1083370250) added to a final concentration of 12.5mM. Cells were grown in 5% CO_2_ balanced with air at 37º C. Fibroblasts and HEK293 cells were maintained by splitting 1:2 or 1:4 when reaching confluence. All experiments in fibroblasts occurred between population doubling 24 and 33. For each experiment, compared data used cells at the same population doubling.

### Cell viability assays

Human neonatal fibroblasts or HEK293 cells were plated onto 24-well plates 1:4 from plates that had just reached approximately 95% confluent and incubated for 48 h for adherence and growth to approximately 50% confluence. These cells were exposed to conditions as detailed below (see paraquat, RSL3, BSO/Fe(III) Citrate). At the end of these exposures, media was removed from wells and replaced with pre-warmed 1X PBS (ThermoFisher 10010023, pH 7.4, containing Hoechst 33342 (Sigma 14533) and ethidium homodimer 1 (Ethd-1, Sigma 46043) at 1 µg/mL and 2 µM, respectively. Cells were incubated in dye solution for 20 min at 37º C. After incubation, cells were imaged on a FLoid (Invitrogen 4471136) inverted fluorescent microscope equipped with brightfield, DAPI (excitation: 390/40 nm, emission 446/33 nm), GFP (excitation 482/18 nm, emission 532/59 nm), and RFP (excitation 586/15 nm, emission 646/68 nm) cubes. Cells staining positively for with Ethd-1 (a cell-impermeant DNA dye, RFP filter cube) were deemed non-viable, while Hoechst 33342 (a cell permeant DNA dye, DAPI filter cube) was used to identify all nuclei. In each assay, a vatiquinone stock solution was prepared by mixing vatiquinone (liquid), measured by weight, into DMSO to 400 µM final concentration.

#### Paraquat

Anhydrous paraquat was solubilized in 1X PBS to a final stock concentration of 1 M. This diluted in culture media to working concentrations of 5 mM and 0.5 mM. Vatiquinone stock solutions (1,000X in DMSO, 125 µM and 500 µM) were diluted in full cell culture media to the appropriate wells to reach the desired concentration (125 nM or 500 nM). Equal DMSO added to non-vatiquinone wells. Viability was assessed (as outlined above) at 24, 48, 72, 96, and 144 h following paraquat treatment in fibroblasts. In HEK293 cells, viability with paraquat with or without vatiquinone was assessed at 48 h.

#### RSL3

Anhydrous RSL3 was solubilized in DMSO to generate a stock solution of 10 mM. Vatiquinone (or DMSO) containing media was added first; after 1 h, media was replaced with media containing 0.2 µM or 2 µM RSL3 for fibroblasts and 2 µM or 10 µM RSL3 for HEK293 cells or vehicle along with vatiquinone or DMSO, and cells were returned to the cell culture incubator. Viability was assessed (as outlined above) at 24 h after the addition of RSL3.

#### BSO/Fe(III)Citrate

Ferric citrate (Fe(III)C) was dissolved in full cell culture media to a stock concentration of 2.5 mM. L-buthionine-(S, R)-sulfoximine (BSO) was dissolved in 1XPBS to a stock concentration of 200 mM. Fe(III)C and BSO were added to cell culture media to prepare media containing 25 µM BSO / 100 µM Fe(III)C for fibroblasts and HEK293 cells. 1 mM and 5 mM BSO / 2.5 mM Fe (III) citrate were used for the HEK293 cells. Vatiquinone or vehicle (DMSO) was added to cells, they were incubated for one hour, then media was replaced with cell media containing the respective concentrations of BSO / Fe(III)C with vatiquinone or vehicle (DMSO). Viability was assessed at 24 and 48 h for HEK293 cells and fibroblasts, respectively, following the addition of BSO/Fe(III)C using the method detailed above.

#### Rotenone

Rotenone (inhibitor of complex I of OXPHOS system) was dissolved in DMSO and was added to fibroblasts and HEK293 cells at concentrations of 0.5 µM and 1 µM with or without vatiquinone treatment. Cells were returned to the incubator and morphology and cell death were assessed at designated timepoints following the treatment.

### Ethics statement on animal use

All experiments were approved by the Institute Animal Care and Use Committee at Seattle Children’s Research Institute (Seattle, WA) under protocols IACUC00611 and IACUC00070. Experiments contain similar numbers of male and female mice of each genotype, numbers are provided in figure legends and supplemental files.

### General mouse husbandry

Mice were kept under standard vivarium conditions at Seattle Children’s Research Institute, with a 12-hour light/dark cycle, with the light phase occurring during the day. Mice were socially housed in Thoren cages with no limits to access to food and water. Chow was supplied ad libitum from hoppers. Cages with mice showing neurological symptoms received additional wetted chow and water in dishes on the bottom of the cage to ensure that food and water accessibility would not become a factor for disease progression and survival. All pups were weaned at age P20 or P21.

In experimental animals, the following health parameters were assessed a minimum of 3 times per week, and on every day when animals received an injection (see below):

#### Body weight/Cachexia

**Fig. 1:**
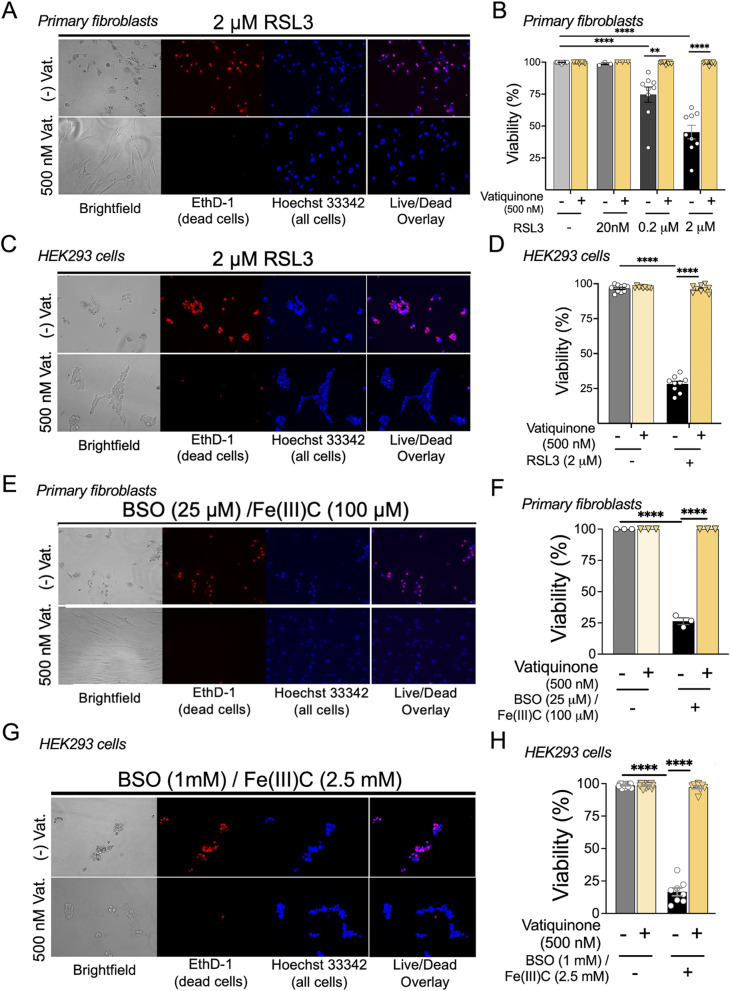
Vatiquinone prevents RSL3 and BSO/Fe(III)C, but not paraquat, induced cell death. (**A**-**B**) RLS toxicity assay in primary human dermal fibroblasts (HDFs, **A**-**B**). Vatiquinone (or DMSO) was added one hour prior to addition of RSL3 and viability assessed by DAPI and EthD-1 staining 24 h after RSL3 was added. (**A**) Representative images of HDFs after 24 h of exposure to 0 or 2 µM RSL3 in the presence or absence of 500 nM vatiquinone. Panels show brightfield, EthD-1 (a viable cell impermeant DNA dye), Hoechst 33,342 (a cell permeant DNA dye), and Hoechst 33,342/EthD-1 overlay (see *Methods*). 2 µM RSL3 induced significant morphologic changes by 24 h which were prevented by vatiquinone (not quantified). RSL3 treatment robustly induced cell death by EthD-1 staining, quantified in (**B**). (**B**) Quantification of cell viability in HDFs treated with RSL3 (or DMSO) with or without the addition of 500 nM vatiquinone, as indicated (see *Methods*). Viability defined as percent of cells (identified by Hoechst 33342) which were not stained with EthD-1. Vatiquinone fully suppressed cell death induced by RSL3. ** *p* < 0.005, ****p < 0.0001 by pairwise t-test. *n* = 9 per group. Two-way ANOVA ****p < 0.0001 for both variables. (**C**-**D**) RLS toxicity assay in HEK293 cells. Vatiquinone (or DMSO) was added one hour prior to addition of RSL3 and viability assessed by DAPI and EthD-1 staining 24 h after RSL3 was added. (**C**) Representative images of HEK293 cells after 24 h of exposure to 0 or 2 µM RSL3 in the presence or absence of 500 nM vatiquinone. Panels as in (**A**). 2 µM RSL3 induced significant morphologic changes by 24 h which fully suppressed by vatiquinone (not quantified). RSL3 treatment robustly induced cell death by EthD-1 staining, quantified in (**D**). (**D**) Quantification of cell viability in HEK293 cells treated with RSL3 (or DMSO) with or without the addition of 500 nM vatiquinone, as indicated (see *Methods*, panel (**B**)). Vatiquinone fully suppressed cell death induced by RSL3. ** *p* < 0.005, ****p < 0.0001 by pairwise t-test. *n* = 9 per group. Two-way ANOVA ****p < 0.0001 for both variables. (**E**-**F**) Impact of vatiquinone on BSO/Fe(III)Citrate induced cell death in HDFs. (**E**) Representative cell viability staining (dyes detailed in (**A**)) from cells treated with BSO and 25 µM BSO / 100 µM Fe(III)C in the presence or absence of 500 nM vatiquinone. BSO + Fe(III)C induced significant morphologic changes which were prevented by vatiquinone (not quantified). (**F**) Cell viability after 24 h of exposure to BSO/Fe(III)C with or without vatiquinone, as indicated. Vatiquinone fully suppressed cell death induced by 25 µM BSO / 100 µM Fe(III)C. ****p < 0.0001 by pairwise t-test. *n* = 9 per group. Two-way ANOVA ****p < 0.0001 for both variables. (**G**-**H**) Impact of vatiquinone on BSO/Fe(III)Citrate induced cell death in HEK293 cells. (**G**) Representative cell viability staining (dyes detailed in (**A**)) from cells treated with BSO and 1 mM BSO / 2.5 mM Fe(III)C in the presence or absence of 500 nM vatiquinone. BSO + Fe(III)C induced significant morphologic changes which were prevented by vatiquinone (not quantified). (**H**) Cell viability after 48 h of exposure to BSO/Fe(III)C with or without vatiquinone, as indicated (see *Methods*, panels (**B**), (**D**), (**E**)). Vatiquinone fully suppressed cell death induced by 25 µM BSO / 100 µM Fe(III)C. ****p < 0.0001 by pairwise t-test. *n* = 18 for no BSO/Fe(III)C, 8 for (+) BSO/Fe(III)C, and 9 for (+) BSO/Fe(III)C/(+) vatiquinone. Two-way ANOVA ****p < 0.0001 for both variables

Cachexia onset (Figs. 1 and 3) is reported as the post-natal day when an individual animal’s weight peaks, i.e. when progressive weight loss starts, as in our prior studies [16–19].

*Forelimb clasping*, a sign of neurologic disease progression, was scored, as previously described [16–19]. As disease progresses in *Ndufs4*(KO)’s, animals can display intermittent/transient improvement of symptoms. Here, we report the age at which animals first present symptom which persist for two or more consecutive days.

#### Survival

The same euthanasia criteria were applied to both the *Ndufs4*(-/-) and *Gpx4* conditional knockout models. Animals were humanely euthanized when they had lost 20% of their body weight from maximum (measured twice consecutively), presented with acute motility or neurologic symptoms perceived to impair ability to access to food or water (immobility, prostrate posture, etc.), or if they were otherwise moribund in appearance (inactive and dehydrated, etc.).

### Gpx4 conditional knockout mice

*Gpx4* floxed allele animals (*Gpx4*(fl/fl)) were obtained from Jackson laboratories (strain 027964). In these animals, exons 2–4 of *Gpx4* are flanked by loxP sites, allowing for conditional or inducible knockout by expression of Cre recombinase. Rosa26CreERT2, which carry a pan-expression tamoxifen inducible Cre cassette, were also purchased from Jackson laboratories (strain 008463). These two lines were bred to generate *Gpx4*(fl/fl)/Rosa26CreERT2(+/+) line. Genotyping of Rosa26CreERT and *Gpx4*(fl/fl) was performed as detailed in *Supplemental Files 1* (‘Gpx4 floxed Genotyping Protocol’) and *Supplemental File 2* (‘Rosa26CreERT2 Genotyping Protocol’). Tamoxifen treatment for Cre induction occurred on P25-P28; animals were injected with 60 µg tamoxifen/g mouse/day on P25, P26, and P27, and 60 µg tamoxifen/g mouse on P28 (see also *Results*). Animals treated with vatiquinone or vehicle for vatiquinone were injected with the drug starting at P21, prior to tamoxifen driven induction of Cre expression. On the days when both tamoxifen and vatiquinone (or vehicle for vatiquinone) were both provided, they were provided as a mixture to minimize the number and total volume of injections. Ataxia was scored by visual assessment with any overtly uncoordinated gait or overt reduction in overall movement leading to a score of ataxia.

As previously reported for this model [4, 20], tamoxifen injections elicited disease in all Gpx4(fl/fl)/Rosa26CreERT2 mice but in none of the Gpx4(WT/WT) or Gpx4(WT/fl) animals (tamoxifen treatment details below). No symptoms were ever observed in Gpx4(fl/fl)/Rosa26CreERT2 mice that were injected with oil only (rather than tamoxifen) or not injected, as has been previously reported.

### Ndufs4(-/-) mice

*Ndufs4*(+/–) mice (originally obtained from the Palmiter laboratory, University of Washington, Seattle, Washington, USA; available through The Jackson Laboratory, strain 027058) were bred to produce *Ndufs4*(-/-) offspring. Mice were weaned at 20–21 days of age and *Ndufs4*(-/-) animals were housed with control littermates for warmth and stimulation. Mice were weighed and health was assessed a minimum of 3 times per week, daily on days when injected. Wetted chow in a dish on the bottom of each cage, and in-cage water bottles, were provided to cages housing *Ndufs4*(-/-) mice following onset of symptoms so that food and water accessibility was not a limiting factor for disease progression and survival. Vatiquinone and vehicle treatments started at P21, weaning. Animals were humanely euthanized when they had lost 20% of their body weight from maximum (measured twice consecutively), on presentation with acute motility or neurologic symptoms perceived to impair ability to access to food or water (immobility, prostrate posture, etc.) or if they were otherwise moribund in appearance (inactive and dehydrated, etc.). As previously reported, the *Ndufs4* deletion is fully recessive: heterozygosity results in no reported phenotypes and no detectable defects in ETC CI activity. Accordingly, “control” cohorts include *Ndufs4*(+/−) and *Ndufs4*(+/+) mice.

### Longitudinal assessments of disease

**Fig. 2:**
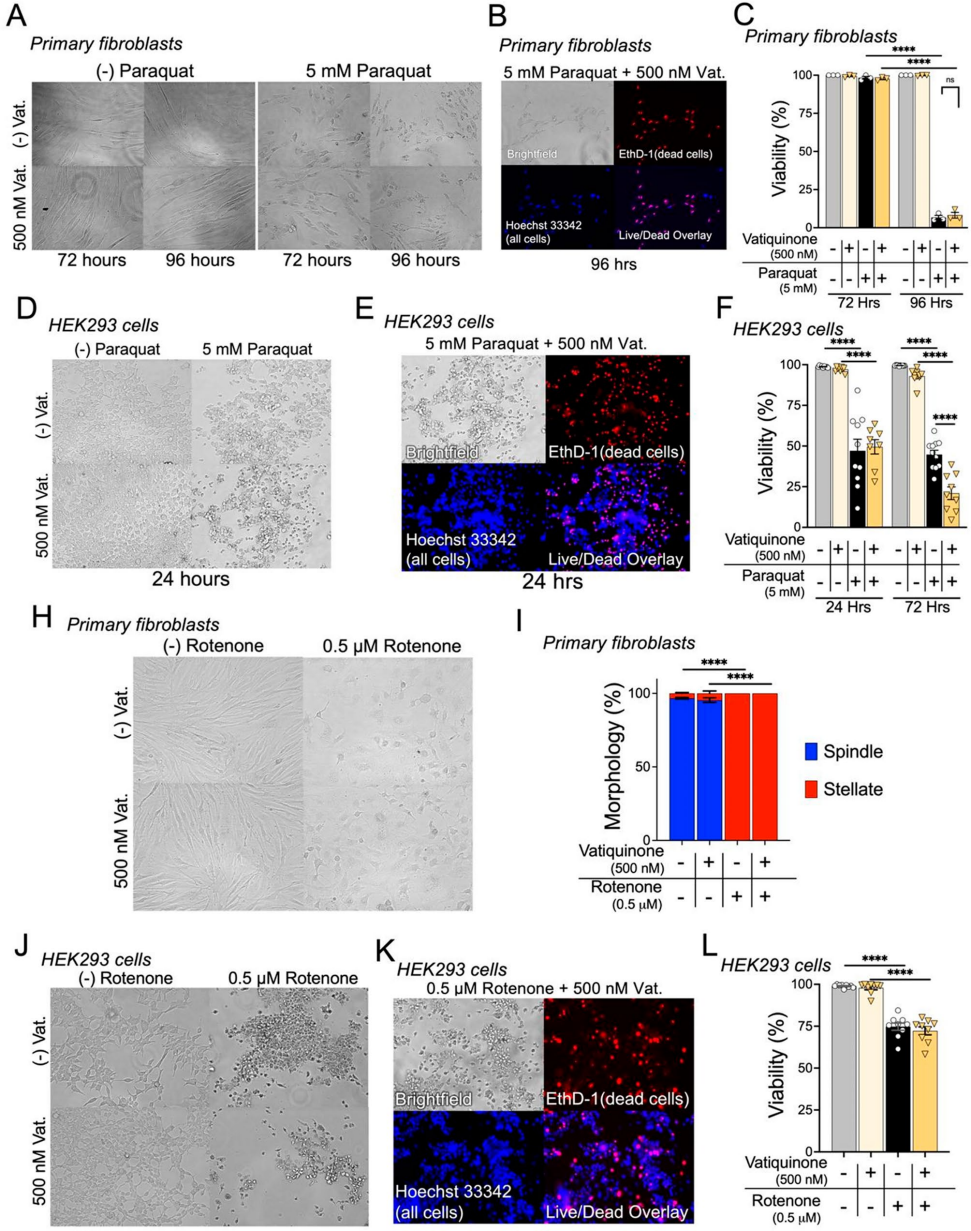
Vatiquinone does not attenuate cellular pathology resulting from mitochondrial ROS or ETC CI inhibition. (**A**-**C**) Assessment of paraquat toxicity and the impact of vatiquinone treatment in primary human dermal fibroblasts. Fibroblasts were treated with 5 mM paraquat in the presence or absence of 500 nM vatiquinone and cell morphology and viability were assessed at 72 and 96 h (see *Methods* for assay details). (**A**) Fibroblasts exposed to 5 mM paraquat show significant morphologic changes by 72 h, which were not impacted by vatiquinone. (**B**-**C**) The majority of paraquat exposed fibroblasts died between 72 and 96 h of paraquat exposure, as assessed by viability staining using EthD-1 and Hoechst 33,342 (see *Methods*, Fig. 1). Vatiquinone had no impact on cell death resulting from paraquat exposure. ****p < 0.0001 by pairwise t-test. n.s.– not significant by pairwise t-test. *n* = 3 per timepoint per treatment. (**D**-**H**) Assessment of paraquat toxicity and the impact of vatiquinone treatment in HEK293 cells. HEK293 cells were treated with 5 mM paraquat in the presence or absence of 500 nM vatiquinone and cell morphology and viability were assessed at 24 and 72 h (see *Methods* for assay details). (**D**) HEK293 cells exposed to 5 mM paraquat show significant morphologic changes by 24 h, including detachment from the culture plate surface. These morphologic changes and detachment were not impacted by vatiquinone. (**E**-**F**) A significant fraction of paraquat exposed HEK293 cells were dead at 24 h of exposure, as assessed by viability staining using EthD-1 and Hoechst 33,342 (see *Methods*, Fig. 1). Vatiquinone had no impact on cell death resulting from paraquat exposure at 24 h. Vatiquinone treatment in combination with paraquat resulted in statistically significantly greater cell death at 72 h of exposure. ****p < 0.0001 by pairwise t-test. *n* = 9 per timepoint per treatment. (**H**-**I**) Assessment of rotenone toxicity and the impact of vatiquinone treatment in primary human dermal fibroblasts. Fibroblasts were treated with 0.5 µM rotenone in the presence or absence of 500 nM vatiquinone and assessed at 48 h (see *Methods* for assay details). (**H**) Fibroblasts exposed to rotenone show significant morphologic changes by 48 h, which were not impacted by vatiquinone. No significant cell death was observed at 48, 72, or 96 h of treatment (data not shown). (**I**) Quantification of morphologic state of fibroblasts exposed for 24 h. The majority of fibroblasts show spindle morphology in the absence of rotenone, and this is unaffected by vatiquinone. All rotenone exposed fibroblasts, whether treated with vatiquinone or not, shifted to a stellate morphology. ****p < 0.0001 by pairwise t-test, *n* = 3 replicates per group. (**J**-**L**) Assessment of rotenone toxicity and the impact of vatiquinone treatment in HEK293 cells. HEK293 cells were treated with 0.5 µM rotenone in the presence or absence of 500 nM vatiquinone and cell morphology and viability were assessed at 48 h (see *Methods* for details). (**J**-**K**) HEK293 cells exposed to 0.5 µM rotenone show significant morphologic changes by 24 h, including detachment from the culture plate surface. These morphologic changes and detachment were not impacted by vatiquinone. (**K**-**L**) Approximately one quarter of rotenone exposed HEK293 cells were dead at 24 h of exposure, as assessed by viability staining using EthD-1 and Hoechst 33,342 (see *Methods*, Fig. 1). Vatiquinone had no impact on cell death resulting from rotenone exposure. ****p < 0.0001 by pairwise t-test, vatiquinone treated groups not significantly different from matched non-vatiquinone groups. *n* = 9 per group

**Fig. 3:**
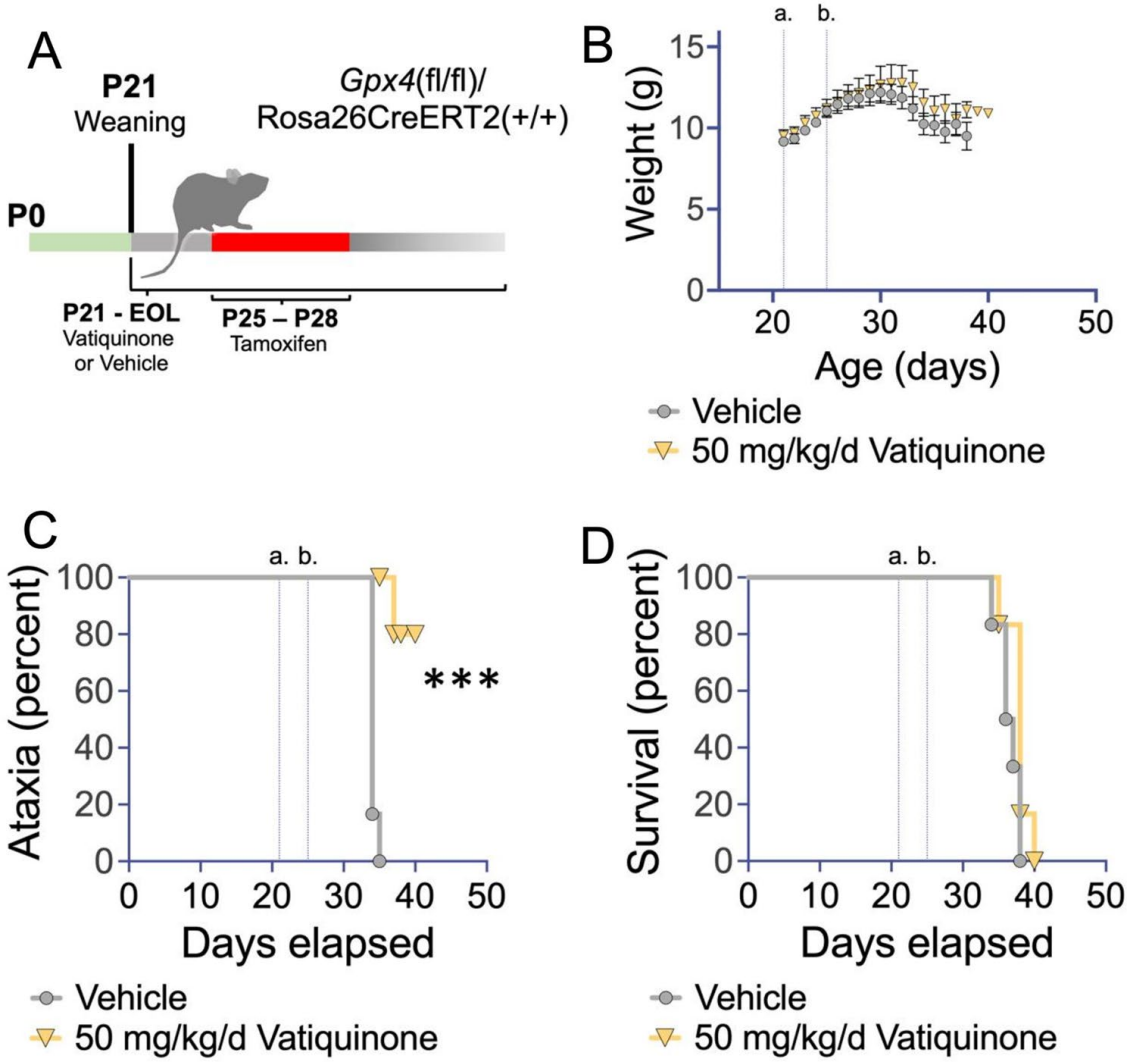
Vatiquinone delays ataxia but not death in an inducible *Gpx4* knockout model. (**A**) Schematic of studies in the *Gpx4*(fl/fl)/Rosa26CreERT2(+/+) inducible model for *Gpx4* deficiency. Treatment with vatiquinone (50 mg/kg/day) or vehicle (see *Methods*) by IP injection began at weaning, P21, and continued until end of life criteria were met. Tamoxifen was administered from P25-P28, provided in one injection with vatiquinone or vehicle (see *Methods*). (**B**) Weight curves of *Gpx4*(fl/fl)/Rosa26CreERT2(+/+) treated with tamoxifen at P25-P28 and vatiquinone or vehicle from P21. Vertical lines indicate weaning and start of vatiquinone or vehicle treatment (a.) and P25, the beginning of Cre induction by tamoxifen (b.). Data are shown as mean with error bars displaying standard error of the mean. There were no significant differences in weight by treatment group at any age. (**C**) Onset of ataxia by visual assessment (see *Methods*). Vatiquinone treated inducible *Gpx4* knockout animals showed a statistically significant delay in the appearance of ataxia (***p < 0.0005 by log-rank test). Vertical lines show weaning (a) and first day of tamoxifen treatment (b) as in (**B**). (**D**) Survival of inducible *Gpx4* deficient animals. Vertical lines show weaning (a) and first day of tamoxifen treatment (b) as in (**B**). Vatiquinone did not alter survival in inducible *Gpx4* deficient mice. (**B**-**C**) *n* = 6 animals per group

Forelimb clasping, a sign of neurologic disease progression, was assessed by visual scoring, as previously described [16–19]. As disease progresses in *Ndufs4*(KO)’s animals display intermittent/transient improvement of symptoms. Here, we simply report whether animals ever presented the symptom for two or more consecutive days (this criterion to minimize spurious reporting). Cachexia onset (Figs. 2 and 3) is defined as the day of life when an individual animal’s weight peaks and progressive weight loss starts, as in our prior studies [17–19, 21].

### Drugs for injection

Vatiquinone stock was prepared by dissolving vatiquinone in sunflower seed oil to a final concentration of 5 µg/uL (w/v), passing through a 0.22 micron sterile filter, aliquoting, and storing aliquots at -20ºC (and protected from light) until use. Vatiquinone treated mice were injected with 10 µL/g of this solution (10 µL/g body weight = 50 µg vatiquinone/g body weight), and vehicle treated mice injected with 10 µL/g of sterile sunflower seed oil only. Injected solutions were warmed to 25–37ºC prior to injection.

Tamoxifen was prepared at 6 mg/mL in 95% (by volume) sunflower seed oil/5% (by volume) ethanol and stored in sterile (0.22 micron filtered) aliquots.

*Cre induction regimen*: On P25-28, Gpx4(fl/fl)/Rosa26CreERT2 animals were injected with 10 µL/g for a dose of 60 µg/g/day. On P29, animals this was diluted 1:1 with 95% sunflower seed oil/5% ethanol for a dose of 30 µg/g (half dose) on the final induction day. In animals treated with vatiquinone and tamoxifen, the injection solution was prepared with both 5 µg/uL vatiquinone and 6 mg/mL (or 3 mg/mL on the last day) tamoxifen in the same mixture, allowing for administration of both compounds in the same injection. Control (no vatiquinone) animals were provided matching vehicle injections on each day.

### Rotarod and rotarod-induced seizures

Mouse rotarod performance was assessed at P30, with seizure incidence noted. Rotarod was performed using a Med Associates ENV-571 M single-lane rotarod. Assays were performed by placing animals onto an already rotating rod and timing latency to fall. Rotation was set to constant 6 rpm (controlled using Med Associates software). Maximum time of each trial was 10 min. For each assay, three trials were performed, with a minimum of 5 min between trials. The greatest latency to fall of the three trials was used for comparisons.

Mice were monitored during each trial for seizure activity. Seizures were identified by any of the symptoms on the Racine behavioral scale or Pinel and Rovner scales, but abnormal oroalimentary movements (dropping of the jaw repeatedly, atypical gnawing or chewing movements), and repeat head nodding were not considered ‘definitive’ for seizure activity and did not lead to trial halt or seizure scoring. Anterior limb clonus (twitching/jumping), dorsal extension/rearing, loss of balance and violent falling (observed when mouse was not on the rotarod), violently running/jumping were considered definitive of seizures and resulted in both a halt to trials that day and recording of a seizure incident. No attempt was made to score seizure severity for these studies– we assessed only presence vs. absence.

## Results

### Vatiquinone suppresses RSL3 and BSO/Fe(III)C induced cell death

To establish the efficacy of our commercially obtained vatiquinone (see *Methods*), we first treated primary human neonatal dermal fibroblasts (HDFs) and HEK293 cells with RSL3 in the presence or absence of vatiquinone (see *Methods*). RSL3 is an inhibitor of the mitochondrial antioxidant enzyme glutathione peroxidase 4, GPX4, and induces ferroptotic cell death *via* this inhibition (see *Introduction)* [12, 13]. Vatiquinone has previously been shown potently suppress RSL3-induced ferroptosis in mitochondrial disease patient cell lines [12].

In HDFs, 0.2 µM and 2 µM RSL3 led to approximately 20% and 50% cell death, respectively, by EthD-1 staining (see *Methods*) at 24 h of exposure (Fig. 1A-B). RSL3-induced cell death was fully suppressed by 500 nM vatiquinone (Fig. 1A-B). Similarly, ~ 75% of HEK293 cells stained positively for EthD-1 after 24 h of exposure to 2 µM RSL3, and cell death was fully suppressed by 500 nM vatiquinone (Fig. 1C-D). These findings are in line with previous reports using vatiquinone provided by BioElectron [12].

To further probe the efficacy of vatiquinone, we next treated fibroblasts and HEK293 cells with L-buthionine-(S, R)-sulfoximine (BSO) and Fe(III)Citrate (Fe(III)C). BSO is an irreversible inhibitor of γ-glutamylcysteine synthetase (γ-GCS) and drives depletion of glutathione by inhibiting glutathione synthesis. This leads to an inhibition of GPX4, as glutathione is a necessary co-factor. When cells are treated with BSO and provided with excess labile iron, which can be added in the form of Fe(III)C, ferroptosis is induced, and vatiquinone has previously been found to potently inhibit BSO/Fe(III)C induced cell death [12, 22]. BSO/Fe(III)C induced significant cell death in both fibroblasts and HEK293 cells and, as with RSL3, vatiquinone robustly prevents BSO/Fe(III)C induced cell death in both cell lines (Fig. 1E-H, see *Methods* for details).

### Vatiquinone has no impact on paraquat-induced cell death

Vatiquinone was identified in screen related to induction of ferroptosis (see *Introduction*). RSL3 and both BSO/Fe(III)C both induce cell death through this pathway. While the known target of vatiquinone (15-LO) suggests specificity for ferroptosis, vatiquinone is often referred to as a general antioxidant, for example described as having ‘up to 10,000 times [the] antioxidant potency’ of CoQ_10_ [23, 24]. However, data assessing the impact of vatiquinone in the setting of other oxidative stressors is limited.

To determine vatiquinone prevents toxicity from oxidative stresses beyond the ferroptosis pathway, we next treated HDFs and HEK293 cells with paraquat. Paraquat (N, N′-dimethyl-4,4′-bipyridinium dichloride or methyl viologen) is a toxin which generates high levels of ROS, including superoxide radicals (O2•−), hydrogen peroxide (H2O2), and hydroxyl radicals (•OH), at mitochondrial ETC CI [25, 26]. Unlike RSL3 and BSO/Fe(III)C, paraquat does not induce ferroptosis, instead driving cell death through intrinsic (mitochondrial) apoptosis [27–29].

5 mM paraquat robustly induced cell death in both fibroblasts (Fig. 2A-C) and HEK293 cells (Fig. 2D-F). In contrast to our findings with RSL3 and BSO/Fe(III)C, vatiquinone had no impact on paraquat induced cell death in either primary fibroblasts or HEK293 cells (Fig. 2A-F). Interestingly, vatiquinone *enhanced* cell death induced by paraquat in HEK293 cells (Fig. 2F, see *Discussion*).

### Vatiquinone has no impact on rotenone-induced cellular injury

Vatiquinone has been trialed in multiple mitochondrial diseases (see *Introduction*, *Discussion*), which are often linked to defects in genes coding for components of the ETC. We next sought to assess whether vatiquinone has any beneficial effects on cellular endpoints resulting from ETC disruption. To accomplish this, we treated HDFs and HEK293 cells with rotenone, a specific chemical inhibitor of ETC CI which is been used to model ETC CI defects in cell and animal systems [30–32].

In fibroblasts, rotenone treatment did not induce cell death (< 1% EthD-1 positivity in all conditions, data not shown), but led to significant changes in cell morphology by 48 h: rotenone treated fibroblasts adopting stellate, versus normal spindle, morphology (Fig. 2H-I). Vatiquinone had no impact on this morphologic change. In HEK293 cells, 0.5 µM rotenone for 48 h resulted in morphologic changes (Fig. 2J) as well as significant cell death by EthD-1 staining (Fig. 2J-L). Vatiquinone had no impact on rotenone-induced cell death.

### Vatiquinone may delay onset of ataxia, but does not extend survival, in Gpx4 deficient mice

As detailed above, GPX4 inhibits ferroptotic cell death, and vatiquinone prevents cell death from toxins which act through inhibition of GPX4. We next sought to establish whether clinically relevant doses of vatiquinone can attenuate disease resulting from GPX4 loss in vivo. To test this, we used an inducible *Gpx4* knockout mouse line by crossing mice carrying an allele of *Gpx4* where exons 2–4 are flanked by loxP sites (Jackson laboratories strain 027964) and a tamoxifen-inducible, whole-body expressed, Cre recombinase (Rosa26CreERT2, Jackson laboratories strain 008463, see *Methods*). This approach has previously been used to study the impact of *Gpx4* loss in vivo [20], with postnatal loss of *Gpx4* causing mitochondrial dysfunction, mitochondrial oxidative damage, apoptosis, neurodegeneration, and death within 2 weeks (see *Discussion*).

Using animals homozygous for both alleles, we treated animals with either vatiquinone or vehicle solution starting at weaning, postnatal day 21 (P21), by daily IP injection; animals were also treated with tamoxifen from P25-28 to induce Cre expression (Fig. 3A, *Methods*). As previously reported, induction of Cre in these mice led to rapid progressive weight loss, onset of ataxia, and mortality within about two weeks (Fig. 3B-D).

Mice were treated with vehicle (oil) only or 50 µg/kg body weight/day (50 mg/kg body weight/day) vatiquinone. This dose was based on that used for clinical trials and efficacy in vitro (see Table 2). 50 mg/kg/day vatiquinone had no effect on survival or weight loss in *Gpx4* deficient mice but, interestingly, appeared to delay the onset of ataxia.

**Table 2:** Vatiquinone doses in clinical trials compared to studies performed here

Clinical trial NCT number (see Table 1)	Approximate maximum daily dosing by weight*
NCT04378075	40 mg/kg/day
NCT04577352	40 mg/kg/day
NCT02352896	15 mg/kg/day
NCT01962363	20 mg/kg/day
NCT02104336	15 mg/kg/day
NCT01728064	20 mg/kg/day
NCT01721733	15 mg/kg/day
NCT01642056	15 mg/kg/day
**Experimental models**	
Cell culture studies	500 nM, ~ 220 µg/kg media
Mouse studies	50 mg/kg/day

* Assumes lowest allowed body mass at threshold for higher dose and median weight for youngest age when no weight criteria provided

### Vatiquinone has no effect on overall disease progression in the Ndufs4(-/-) mouse model of Leigh syndrome, but may reduce seizures

**Fig. 4:**
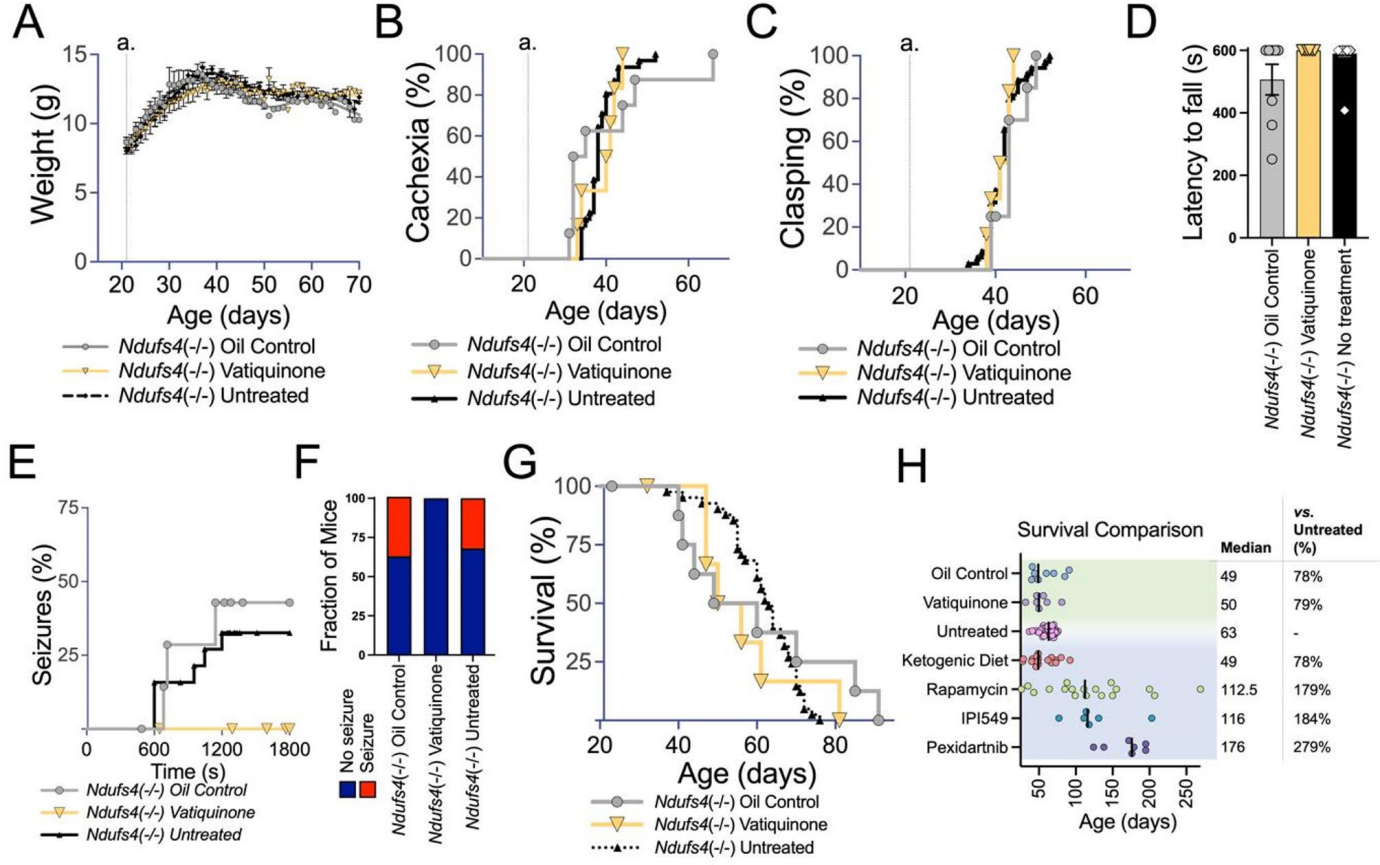
Vatiquinone does not impact overall disease or survival but may prevent seizures in the *Ndufs4*(-/-) model of Leigh syndrome. (**A**) Weights of untreated, vatiquinone treated, or vehicle (oil) only treated *Ndufs4*(-/-) mice. Data are shown as mean with standard error of the mean. No significant differences in weight were observed at any age. Weaning and the start of treatments occur at P21 (line a.). (**B**) Onset of cachexia in untreated *Ndufs4*(-/-) mice and *Ndufs4*(-/-) mice treated with vatiquinone or vehicle (oil) only. Cachexia onset is defined as the age in each individual animal when weight peaks and progressive weight loss begins (see (**A**) for overall weight trajectory for *Ndufs4*(-/-) mice). Weaning and the start of treatments occur at P21 (line a.). Vatiquinone had no significant effect when compared to either vehicle (oil) or untreated cohorts. (**C**) Onset of forelimb clasping in untreated *Ndufs4*(-/-) mice and *Ndufs4*(-/-) mice treated with vatiquinone or vehicle (oil) only. Vatiquinone had no significant effect when compared to either vehicle (oil) or untreated cohorts. Weaning and the start of treatments occur at P21 (line a.). (**D**) Rotarod performance at P30, reported as the best of three trials with a maximum trial time of 600 s (see *Methods*). Treatment groups did not significantly differ, but there is no overt defect in performance at this age; rotarod trials from this age are used to assess seizure frequency (see (**E**-**F**)). (**E**) Seizure incidence as a function of overall time on the rotarod at P30 in untreated *Ndufs4*(-/-) mice and *Ndufs4*(-/-) mice treated with vatiquinone or vehicle (oil) only. Differences did not reach statistical significance (log-rank test) in these limited cohorts, but vatiquinone treated animals did not present with seizures in this assay. (**F**) Overall seizure incidence in the P30 rotarod assay (panels (**D**-**E**)). Differences did not reach statistical significance in these limited cohorts (Fisher’s exact test *p* = 0.156 vatiquinone vs. summed control treatments), but no seizures were observed in vatiquinone treated animals. (**D**-**F**) *n* = 16 (untreated), 8 (vehicle treated), and 6 (vatiquinone treated). (**G**) Survival of untreated *Ndufs4*(-/-) mice and *Ndufs4*(-/-) mice treated with vatiquinone or vehicle (oil) only. No significant differences were observed between treatment groups (log-rank test). (**H**) Survival data from animals in this study (green highlighted region of plot) and additional representative results from other small-molecule intervention trials performed by our group including ketogenic diet [17], 8 mg/kg/day rapamycin by IP injection [16], 100 mg/kg/day IPI-549 orally in chow [19], and 300 mg/kg/day pexidartinib orally in chow [19]. IPI549 and pexidartinib studies occurred partly in tandem with the vatiquinone trials, and the untreated *Ndufs4*(-/-) mouse cohort spanned these trials. Rapamycin, IPI-549, and pexidartinib were found to significantly increase survival, while ketogenic diet had no significant effect (see cited literature for details). Vatiquinone similarly has no significant effect on survival in the *Ndufs4*(-/-). (A-G) *n* = 9 oil treated and 7 vatiquinone treated *Ndufs4*(-/-) animals per dataset

To test the efficacy of vatiquinone in the context of Leigh syndrome, we used the *Ndufs4*(-/-) mouse model of the disease. *Ndufs4*(-/-) animals were treated with 50 mg/kg/day vatiquinone or vehicle solution only starting at weaning, P21, by daily IP injection (see *Methods*). This approach has been effective in other pharmacologic studies in this model, including our own prior work (see *Discussion* and Fig. 4H). Onset of weight loss (cachexia) and overall progressive weight decline from disease onset were not impacted by either vehicle (oil) treatment or by vatiquinone (Fig. 3A-B). Similarly, neither the vehicle nor vatiquinone impacted the age of onset of forelimb clasping, a visually scored symptom of neurologic disease or mortality (Fig. 4C).

*Ndufs4*(-/-) performance on a rotarod assay is not significantly impaired at P30 (Fig. 4D), but the rotarod assay at this age can be used to assess exercise-induced seizure incidence [17]. Overall exercise-induced seizure incidence and time to seizure were the same in oil (vehicle) treated and untreated *Ndufs4*(-/-) mice, but vatiquinone appeared to suppress exercise-induced seizures at this age (Fig. 4E-F, see *Discussion* regarding statistical significance).

Survival of *Ndufs4*(-/-) animals was not significantly impacted by either the vehicle (oil) or by 50 mg/kg/day vatiquinone (Fig. 4G-H).

## Discussion

Vatiquinone/EPI-743 was first identified in a small molecule screen for compounds which prevent cell death caused by the glutathione synthesis inhibitor L-buthionine-(S, R)-sulfoximine (BSO) [23]. These benefits have recently been attributed to inhibition of 15-lipoxygenase (15-LO), which generates the oxidized lipid product 15-hydroxyeicosatetraenoic acid when glutathione levels are low or GPX4 is inactivated [12]. Here, in two cell lines, we have reproduced these findings, showing that vatiquinone prevents cell death from GPX-4 inhibition or glutathione depletion with iron overload (see Fig. 1). In these conditions, vatiquinone appears to be potent, providing robust benefits to multiple cell lines.

In contrast, we find that vatiquinone has no impact on paraquat-induced cell death *or* rotenone-induced cellular pathology in either of two cell lines, indicating that vatiquinone is not effective as a general antioxidant, mitochondrial antioxidant, or as a treatment for cellular consequences of ETC CI dysfunction. These cell-based findings suggest that vatiquinone benefits are probably limited to perturbations of the GPX4/15-LO axis. Our data do not support the notion that vatiquinone would benefit conditions defined by generalized mitochondrial oxidative stress, unless 15-LO mediated cell death has been observed. That scenario may include FA, genetic defects in GPX4, and other factors involved glutathione redox or iron homeostasis, but no current evidence supports the idea that 15-LO is involved in the pathobiology of mitochondrial diseases as a group or Leigh syndrome in particular.

Primary fibroblasts and HEK293 cells provide two very distinct cell types and together suggest that our cellular findings are not cell-type specific. However, we cannot rule out the possibility that unique responses to one or more of these toxins, or vatiquinone, may occur in highly differentiated cell types *in vivo.*

### GPX4 deficiency

Defects in GPX4 can cause Sedaghatian-type spinal metaphyseal dysplasia (SSMD), a severe genetic disease characterized by metaphyseal chondrodysplasia, cardiovascular disease, and neurologic defects [4]. SSMD mutations have been shown to impact enzymatic activity of GPX4. The *Gpx4* conditional knockout mouse model has been used to study disease arising from GPX4 defects, with Cre leading to the accumulation of mitochondrial (but not whole-cell) 4-hydroxynonenal (4-HNE, a product of ROS damage), a reduction in mitochondrial complex I activity and ATP generation capacity, overt cell death (including neurons), and animal death within 5–15 days [20]. Neuron specific knockout leads to a nearly identical rate of death [33]. It was claimed that high dose vitamin E delayed disease, but the extremely small effect (1 day delay in mortality) and lack of any statistical comparisons does not support that conclusion. An examination of the data indicates vitamin E is not effective against GPX4 deficiency.

Given the robust benefits of vatiquinone in the setting of *Gpx4* inhibition in cultured cells, we found it surprising that the compound had no effect on survival in the inducible knockout mouse. One possibility is that total loss of Gpx4 has effects beyond induction of cell death via 15-LO; this would seem to suggest Gpx4 has critical functions beyond ferroptosis. Perhaps perturbation of Gpx4 function leads to controlled cell death via ferroptosis, while complete loss drives additional forms of cell death due to severe oxidative stress. In this scenario, blocking ferroptosis alone may simply be insufficient to counter Gpx4 loss.

Gpx4 loss in mice does appear more severe than SSMD-causing GPX4 mutations in humans as loss in mice causes embryonic lethality or rapid death after postnatal deletion. Accordingly, it is unclear whether *Gpx4* null mice are a useful disease model for SSMD. Intriguingly, ataxia onset was significantly attenuated by vatiquinone in this cohort. These observations will need to be validated, and histologic analysis of neuronal loss performed, to confirm these findings. Future studies using the neuron-specific *Gpx4* deficient model may provide additional insight into the role of 15-LO mediated neuronal death or dysfunction in neurologic sequelae resulting from *Gpx4* loss.

### Leigh syndrome

A number of clinical trials focus on vatiquinone in Leigh syndrome (see below, Table 1). In contrast to SSMD, a robust a well-established mammalian model for Leigh syndrome is available, the *Ndufs4*(-/-) mouse. This animal shares a causal genetic defect with a subset of human Leigh syndrome patients (complete loss of NDUFS4) and displays the major sequelae of the human disease. In particular, the animal model develops progressive, symmetric, neuroinflammatory brain lesions, a defining clinical feature of Leigh syndrome. Multiple pre-clinical interventions have been found to significantly attenuate disease in the *Ndufs4*(-/-). Among these are inhibition of mTOR or PI3Kγ, chronic mild hypoxia at 11% oxygen, which prevents and reverse symptoms, and the CSF1R inhibitor pexidartinib, which prevents disease in the *Ndufs4*(-/-) but is itself toxic in chronic use. Against this backdrop of effective interventions, 50 mg/kg/day vatiquinone had no impact on overall disease onset and progression (see Fig. 3H).

Our findings are not without caveats. *Ndufs4*(-/-) mouse model is considered the premier model for Leigh syndrome, and currently there are no other established mouse models of this disease. Given the overall conservation of *Ndufs4-* related disease from mice to humans it is thought likely that disease mechanisms, and interventions, will be conserved. Critically, however, as LS can be caused by defects in any of over 110 distinct genes (see *Introduction*). The clinical entity LS may actually represent multiple overlapping diseases with shared clinical features. Accordingly, findings in the *Ndufs4*(-/-) mouse may be relevant to only a subset of LS patients– perhaps those with similar defects in the ETC. A better understanding of the pathobiology of LS arising from different genetic causes is needed.

### Seizures

Exercise-induced seizures occur in *Ndufs4*(-/-) at P30 are reduced by therapies including rapamycin and a ketogenic diet [17]. Similar to dietary ketosis, vatiquinone failed to alter overall disease course or extend survival but appears, in our limited cohort, to suppress these seizures (Fig. 3E-F). This is intriguing in light of the MIT-E trial, which failed to achieve primary endpoints but, according to a press release, significantly reduced seizure incidence in the subset of patients with Leigh syndrome (NCT04378075, see Table 1) [12] (June 29, 2023 press release from PTC Therapeutics). Together, clinical and pre-clinical data seem to indicate that 15-LO may be a viable therapeutic target for seizures in Leigh syndrome.

Any mechanistic link between 15-LO and seizures is not immediately clear. Furthermore, our limited supply of vatiquinone allowed (see *Methods*) did not allow for animal numbers sufficient to reach statistical significance; our observations are interesting, but highly preliminary. Follow-up studies should consider the impact of vatiquinone in the GAD2-specific *Ndufs4*(-/-) model, which presents with fatal mitochondrial epilepsy but not progressive neuroinflammatory CNS lesions [34, 35]. This will enable study of seizures in the absence of other, complicating, disease sequelae.

### Clinical significance and ethical perspectives

In the US alone, there have been 19 clinical trials of vatiquinone. Of these, 13 target mitochondrial disease, with six targeting Leigh syndrome either as a component of mitochondrial disease overall (NCT05218655, NCT04378075, NCT01370447, NCT01642056) or as a specific clinical indication (NCT02352896, NCT01721733)(see Table 1). The earliest of these trials dates to 2013. However, as of this writing, results have only been posted to trials.gov for one study (NCT01642056). This trial targeted children with mitochondrial disease and failed to achieve its primary outcomes. Press reports indicate that four additional studies have failed to achieve primary outcomes.

In smaller studies, some promising results have been reported, but these have lacked rigor and sufficient sample size for confident analysis. Three case-reports testing vatiquinone in Leigh syndrome with ten, four, and one patient, respectively, found vatiquinone to be well-tolerated [23, 36, 37]. These studies report some indications suggesting the drug might modestly impact disease progression, but the cohorts are too small, clinical outcomes too unrefined, and benefits to weak for confidence. Additionally, the initial reports from 2012 lack any published follow-up.

Clinical evidence supporting the efficacy of vatiquinone in Leigh syndrome, and mitochondrial disease broadly, has failed to materialize. Nevertheless, anecdotal evidence persists, and our experiences with patient groups indicate that many believe this compound represents a major advance in treating mitochondrial disease. Disease course is known to be highly variable in mitochondrial disease; unfortunately, ebbs in clinical progression are often viewed as linked to any therapy being provided. Misrepresentation of vatiquinone in the scientific literature (for example, as an ‘antioxidant’, ‘scavenger’, or compound that ‘generates glutathione’, and statements that ‘EPI-743 works by improving the regulation of cellular energy metabolism’) greatly exacerbate these issues (citations intentionally omitted). The robust benefits of vatiquinone against ferroptosis are supported by our data, but the compound appears to be highly specific to that pathway.

Rodents lack cytochrome P450 isoform 3A4, CYP3A4, which is the major enzyme involved in vatiquinone metabolism in human, and vatiquinone has been shown to persist at significantly higher plasma levels in rodents compared to humans [38, 39]. We also cannot completely rule out the possibility that a significantly higher dose might provide some benefit, the dose we’ve tested is consistent with human trials and cell-based experiments. We also cannot rule out the possibility that 50 mg/kg/day vatiquinone has some very small effect on survival in the *Ndufs4*(-/-), but such an effect would appear to lack clinical significance.

The failure of 50 mg/kg/day vatiquinone to alter disease course in the *Ndufs4*(-/-) model has implications for future clinical trials. These findings also raise serious questions regarding the ethics of testing experimental compounds in rare disease patients when the mechanism of action is unknown (15-LO was only recently identified as the target of vatiquinone) and there has been no testing in relevant preclinical animal models. Preliminary clinical and pre-clinical evidence suggests vatiquinone may benefits mitochondrial seizures, and cell-based data indicate vatiquinone should benefit settings of increased 15-LO mediated cell death, but our data do not support the notion that vatiquinone might be effective in mitochondrial diseases as a broad clinical group.

Our observations bring to light the lack of reported results from relevant trials (see Table 1). As mitochondrial disease progression is highly varied, off-label, small cohort, and uncontrolled trials can easily result in overly optimistic views of a candidate agent. Speedy reporting of results from controlled trials, whether positive or negative, should be a priority. Populating trials is difficult in rare diseases; testing interventions without robust rationale results in missed opportunities for the patients who could have been involved in other trials and for progress in understanding and treating mitochondrial disease.

Since the first trial of vatiquinone started in 2012, significant progress has been made in identifying therapeutic targets in the mouse model for LS. Most notably, chronic mild hypoxia and immune targeting show significant promise [16, 18, 40–45]. We believe that strategies showing efficacy in a robust animal models should be prioritized over those lacking in vivo evidence, and agents with known mechanism of action should be prioritized over those whose targets are unknown. Finally, the clinical data suggests that target population should be more carefully selected according to the *precise* mode of action of the drug. This, of course, depends on knowing the target of a given compound.

## Data Availability

All data are available within the article.

## References

[1] Schlieben LD, Prokisch H. The dimensions of primary mitochondrial disorders. Front Cell Dev Biol. 2020;8:600079. 33324649 10.3389/fcell.2020.600079 PMC7726223

[2] Newman NJ, Yu-Wai-Man P, Biousse V, Carelli V. Understanding the molecular basis and pathogenesis of hereditary optic neuropathies: towards improved diagnosis and management. Lancet Neurol. 2023;22:172–88. 36155660 10.1016/S1474-4422(22)00174-0

[3] Rahman S. Leigh syndrome. Handb Clin Neurol. 2023;194:43–63. 36813320 10.1016/B978-0-12-821751-1.00015-4

[4] Xie Y, Kang R, Klionsky DJ, Tang D. GPX4 in cell death, autophagy, and disease. Autophagy. 2023;19:2621–38. 37272058 10.1080/15548627.2023.2218764 PMC10472888

[5] Enns GM, Cohen BH. Clinical trials in mitochondrial disease: an update on EPI-743 and RP103. J Inborn Errors Metabolism Screen. 2017;5:2326409817733013.

[6] Kaplan J. Friedreich’s ataxia is a mitochondrial disorder. Proc Natl Acad Sci U S A. 1999;96:10948–9. 10500103 10.1073/pnas.96.20.10948 PMC34221

[7] Borchert A, et al. The role of phospholipid hydroperoxide glutathione peroxidase isoforms in murine embryogenesis. J Biol Chem. 2006;281:19655–64. 16684775 10.1074/jbc.M601195200

[8] Amtmann D, Gammaitoni AR, Galer BS, Salem R, Jensen MP. The impact of TK2 deficiency syndrome and its treatment by nucleoside therapy on quality of life. Mitochondrion. 2023;68:1–9. 36374792 10.1016/j.mito.2022.10.003

[9] Bermejo-Guerrero L, et al. Remarkable clinical improvement with oral nucleoside treatment in a patient with adult-onset TK2 deficiency: a case report. Mitochondrion. 2024;76:101879. 38599303 10.1016/j.mito.2024.101879

[10] Salviati L, Trevisson E, Agosto C, Doimo M, Navas P et al. in *GeneReviews((R))*, M. P. Adam Eds. (Seattle (WA), 1993). 28125198

[11] Tarnopolsky MA. The mitochondrial cocktail: rationale for combined nutraceutical therapy in mitochondrial cytopathies. Adv Drug Deliv Rev. 2008;60:1561–7. 18647623 10.1016/j.addr.2008.05.001

[12] Kahn-Kirby AH, et al. Targeting ferroptosis: a novel therapeutic strategy for the treatment of mitochondrial disease-related epilepsy. PLoS ONE. 2019;14:e0214250. 30921410 10.1371/journal.pone.0214250 PMC6438538

[13] Sekhar KR, et al. Glutathione peroxidase 4 inhibition induces ferroptosis and mTOR pathway suppression in thyroid cancer. Sci Rep. 2022;12:19396. 36371529 10.1038/s41598-022-23906-2 PMC9653479

[14] Cozza G, et al. Glutathione peroxidase 4-catalyzed reduction of lipid hydroperoxides in membranes: the polar head of membrane phospholipids binds the enzyme and addresses the fatty acid hydroperoxide group toward the redox center. Free Radic Biol Med. 2017;112:1–11. 28709976 10.1016/j.freeradbiomed.2017.07.010

[15] Tsubouchi K, et al. Involvement of GPx4-Regulated lipid peroxidation in idiopathic pulmonary fibrosis pathogenesis. J Immunol. 2019;203:2076–87. 31534007 10.4049/jimmunol.1801232

[16] Johnson SC, et al. mTOR inhibition alleviates mitochondrial disease in a mouse model of Leigh syndrome. Science. 2013;342:1524–8. 24231806 10.1126/science.1244360 PMC4055856

[17] Bornstein R, et al. Differential effects of mTOR inhibition and dietary ketosis in a mouse model of subacute necrotizing encephalomyelopathy. Neurobiol Dis. 2022;163:105594. 34933094 10.1016/j.nbd.2021.105594 PMC8770160

[18] Hanaford AR et al. Peripheral macrophages drive CNS disease in the Ndufs4(-/-) model of Leigh syndrome. *Brain Pathol*, e13192 (2023). 10.1111/bpa.13192 PMC10580015 37552802

[19] Stokes JC et al. Leukocytes mediate disease pathogenesis in the Ndufs4(KO) mouse model of Leigh syndrome. JCI Insight 7, (2022). 10.1172/jci.insight.156522 PMC8983133 35050903

[20] Yoo SE, et al. Gpx4 ablation in adult mice results in a lethal phenotype accompanied by neuronal loss in brain. Free Radic Biol Med. 2012;52:1820–7. 22401858 10.1016/j.freeradbiomed.2012.02.043 PMC3341497

[21] Spencer KA et al. Volatile anaesthetic toxicity in the genetic mitochondrial disease Leigh syndrome. Br J Anaesth, (2023). 10.1016/j.bja.2023.08.009 PMC10636522 37770252

[22] Ju J, Song YN, Wang K. Mechanism of ferroptosis: a potential target for Cardiovascular diseases Treatment. Aging Dis. 2021;12:261–76. 33532140 10.14336/AD.2020.0323 PMC7801281

[23] Enns GM, et al. Initial experience in the treatment of inherited mitochondrial disease with EPI-743. Mol Genet Metab. 2012;105:91–102. 22115768 10.1016/j.ymgme.2011.10.009

[24] Tinker RJ, Lim AZ, Stefanetti RJ, McFarland R. Current and emerging clinical treatment in mitochondrial disease. Mol Diagn Ther. 2021;25:181–206. 33646563 10.1007/s40291-020-00510-6 PMC7919238

[25] Castello PR, Drechsel DA, Patel M. Mitochondria are a major source of paraquat-induced reactive oxygen species production in the brain. J Biol Chem. 2007;282:14186–93. 17389593 10.1074/jbc.M700827200 PMC3088512

[26] Cocheme HM, Murphy MP. Complex I is the major site of mitochondrial superoxide production by paraquat. J Biol Chem. 2008;283:1786–98. 18039652 10.1074/jbc.M708597200

[27] Jang YJ, et al. Paraquat induces apoptosis through a Mitochondria-Dependent pathway in RAW264.7 cells. Biomol Ther (Seoul). 2015;23:407–13. 26336579 10.4062/biomolther.2015.075 PMC4556199

[28] Yang W, Tiffany-Castiglioni E. Paraquat-induced apoptosis in human neuroblastoma SH-SY5Y cells: involvement of p53 and mitochondria. J Toxicol Environ Health A. 2008;71:289–99. 18253895 10.1080/15287390701738467

[29] Ramachandiran S, Hansen JM, Jones DP, Richardson JR, Miller GW. Divergent mechanisms of paraquat, MPP+, and rotenone toxicity: oxidation of thioredoxin and caspase-3 activation. Toxicol Sci. 2007;95:163–71. 17018646 10.1093/toxsci/kfl125

[30] Byrnes J, et al. Pharmacologic modeling of primary mitochondrial respiratory chain dysfunction in zebrafish. Neurochem Int. 2018;117:23–34. 28732770 10.1016/j.neuint.2017.07.008 PMC5773416

[31] Innos J, Hickey MA. Using rotenone to Model Parkinson’s Disease in mice: a review of the role of Pharmacokinetics. Chem Res Toxicol. 2021;34:1223–39. 33961406 10.1021/acs.chemrestox.0c00522

[32] Won JH, Park S, Hong S, Son S, Yu JW. Rotenone-induced Impairment of Mitochondrial Electron Transport Chain Confers a selective Priming Signal for NLRP3 inflammasome activation. J Biol Chem. 2015;290:27425–37. 26416893 10.1074/jbc.M115.667063 PMC4646374

[33] Chen L, Hambright WS, Na R, Ran Q. Ablation of the Ferroptosis Inhibitor Glutathione Peroxidase 4 in neurons results in Rapid Motor Neuron Degeneration and Paralysis. J Biol Chem. 2015;290:28097–106. 26400084 10.1074/jbc.M115.680090 PMC4653669

[34] Johnson SC, et al. Regional metabolic signatures in the Ndufs4(KO) mouse brain implicate defective glutamate/alpha-ketoglutarate metabolism in mitochondrial disease. Mol Genet Metab. 2020;130:118–32. 32331968 10.1016/j.ymgme.2020.03.007 PMC7272141

[35] Bolea I et al. Defined neuronal populations drive fatal phenotype in a mouse model of Leigh syndrome. Elife 8, (2019). 10.7554/eLife.47163 PMC6731060 31403401

[36] Kouga T, et al. Japanese Leigh syndrome case treated with EPI-743. Brain Dev. 2018;40:145–9. 28916229 10.1016/j.braindev.2017.08.005

[37] Martinelli D, et al. EPI-743 reverses the progression of the pediatric mitochondrial disease–genetically defined Leigh Syndrome. Mol Genet Metab. 2012;107:383–8. 23010433 10.1016/j.ymgme.2012.09.007

[38] Ma J, et al. Absorption, distribution, metabolism and excretion of (14)C-vatiquinone in rats, dogs, and human subjects. Xenobiotica. 2023;53:396–411. 37552765 10.1080/00498254.2023.2245459

[39] Lee L, et al. Effect of Itraconazole, a CYP3A4 inhibitor, and Rifampin, a CYP3A4 inducer, on the pharmacokinetics of Vatiquinone. Clin Pharmacol Drug Dev. 2024;13:1283–90. 39133029 10.1002/cpdd.1461 PMC11609057

[40] Hanaford A, Johnson SC. The immune system as a driver of mitochondrial disease pathogenesis: a review of evidence. Orphanet J Rare Dis. 2022;17:335. 36056365 10.1186/s13023-022-02495-3 PMC9438277

[41] Johnson SC, et al. mTOR inhibitors may benefit kidney transplant recipients with mitochondrial diseases. Kidney Int. 2019;95:455–66. 30471880 10.1016/j.kint.2018.08.038 PMC6389356

[42] Johnson SC, et al. Dose-dependent effects of mTOR inhibition on weight and mitochondrial disease in mice. Front Genet. 2015;6:247. 26257774 10.3389/fgene.2015.00247 PMC4510413

[43] Bornstein R, et al. Differential effects of mTOR inhibition and dietary ketosis in a mouse model of subacute necrotizing encephalomyelopathy. Neurobiol Dis. 2021;163:105594. 34933094 10.1016/j.nbd.2021.105594 PMC8770160

[44] Ferrari M, et al. Hypoxia treatment reverses neurodegenerative disease in a mouse model of Leigh syndrome. Proc Natl Acad Sci U S A. 2017;114:E4241–50. 28483998 10.1073/pnas.1621511114 PMC5448167

[45] Jain IH, et al. Hypoxia as a therapy for mitochondrial disease. Science. 2016;352:54–61. 26917594 10.1126/science.aad9642 PMC4860742

